# OPA1 deficiency impairs oxidative metabolism in cycling cells, underlining a translational approach for degenerative diseases

**DOI:** 10.1242/dmm.050266

**Published:** 2023-09-20

**Authors:** Aurélie M. C. Millet, Corentin Coustham, Camille Champigny, Marlène Botella, Christine Demeilliers, Anne Devin, Anne Galinier, Pascale Belenguer, Joel Bordeneuve-Guibé, Noélie Davezac

**Affiliations:** ^1^ Research Center on Animal Cognition (CRCA), Center for Integrative Biology (CBI), Toulouse University, CNRS, UPS, 31400 Toulouse, France; ^2^ ISAE-SUPAERO, Toulouse University, 31400 Toulouse, France; ^3^ Université Grenoble Alpes, Inserm U1055, LBFA, 38000 Grenoble, France; ^4^ Laboratoire Métabolisme Energétique Cellulaire IBGC du CNRS, 1 rue Camille Saint Saëns, 33077 Bordeaux cedex, France; ^5^ RESTORE – Université de Toulouse, CNRS ERL5311, EFS, INP-ENVT, Inserm U1031, UPS, Bâtiment INCERE, 4bis avenue Hubert Curien, 31100 Toulouse, France

**Keywords:** Mathematical model, Mitochondria, Neurodegenerative disease, Oxidative metabolism

## Abstract

Dominant optic atrophy is an optic neuropathy with varying clinical symptoms and progression. A severe disorder is associated with certain *OPA1* mutations and includes additional symptoms for >20% of patients. This underscores the consequences of OPA1 mutations in different cellular populations, not only retinal ganglionic cells. We assessed the effects of OPA1 loss of function on oxidative metabolism and antioxidant defences using an RNA-silencing strategy in a human epithelial cell line. We observed a decrease in the mitochondrial respiratory chain complexes, associated with a reduction in aconitase activity related to an increase in reactive oxygen species (ROS) production. In response, the NRF2 (also known as NFE2L2) transcription factor was translocated into the nucleus and upregulated SOD1 and GSTP1. This study highlights the effects of OPA1 deficiency on oxidative metabolism in replicative cells, as already shown in neurons. It underlines a translational process to use cycling cells to circumvent and describe oxidative metabolism. Moreover, it paves the way to predict the evolution of dominant optic atrophy using mathematical models that consider mitochondrial ROS production and their detoxifying pathways.

## Teaser

**Summary:** This study highlights the effects of OPA1 deficiency on oxidative metabolism in replicative cells and the use of a mathematical model in a translational process in a neurodegenerative context.

## INTRODUCTION

Dominant optic atrophy (DOA) is characterized by moderate-to-severe loss of visual acuity with insidious onset in early childhood (Amati-Bonneau et al., 2009; Lenaers et al., 2012; Yu-Wai-Man et al., 2011). This disease primarily affects retinal ganglion cells (RGCs), of which the axons forming the optic nerve degenerate. The estimated prevalence is between 1:10,000 in Denmark and 1:50,000 worldwide. There is considerable inter- and intra-familial variability, and penetrance may be as low as 40%. Different studies have shown certain *OPA1* mutations to be associated with a severe multi-systemic disorder (DOA^+^ syndrome) (Amati-Bonneau et al., 2008; Cohn et al., 2007; Yu-Wai-Man et al., 2010; Zeviani, 2008). DOA^+^ patients present additional neurological complications, such as ataxia, sensorineural deafness, sensory–motor neuropathy, progressive external ophthalmoplegia, parkinsonism and myopathy. Altogether, these findings highlight the widespread deleterious consequences of OPA1 mutations, not only for RGCs but also for other cell populations (Barboni et al., 2013; Chao de la Barca et al., 2016; Mackey and Trounce, 2010; Spinazzi et al., 2008; Zeviani, 2008). There is currently no effective treatment for this complex disease.

Most DOA patients (∼75%) harbour mutations in the *OPA1* gene, which encodes a mitochondrial GTPase (Delettre et al., 2000) localized in the inter-membrane space and anchored to the mitochondrial inner membrane (Griparic et al., 2004; Ishihara et al., 2006; Olichon et al., 2002; Satoh et al., 2003). Most *OPA1* mutations result in premature termination, with the ensuing OPA1 haploinsufficiency a major pathogenic mechanism (Amati-Bonneau et al., 2009). OPA1 protein has been shown to be involved in fusion of the mitochondrial inner membrane and the structure of the cristae in various cell lines. Fusion and fission of mitochondria control mitochondrial morphology and regulate the major mitochondrial functions (Bertholet et al., 2016). These processes also contribute to the quality control of the organelle. Through its role in organizing the structure of the cristae, OPA1 sequesters cytochrome c inside the cristae and thus exerts an anti-apoptotic function. Data on skin fibroblasts, muscle and lymphoblasts from DOA patients globally show altered mitochondrial morphology and energetics, as well as increased sensitivity to apoptosis (Alavi et al., 2009; Chevrollier et al., 2008; Olichon et al., 2006; Spinazzi et al., 2008; Yu-Wai-Man et al., 2011; Zanna et al., 2008). However, there are numerous contradictory reports concerning the existence and nature of energetic defects in DOA patients; thus, the extent to which these processes contribute to the pathogenesis of DOA is still unknown. Furthermore, two invertebrate DOA models and, more recently, a mammalian DOA model have linked the critical generation of reactive oxygen species (ROS) to OPA1 dysfunction (Millet et al., 2017, 2016; Shahrestani et al., 2009; Tang et al., 2009).

We previously developed a deterministic mathematical model able to predict the ROS production of complex I of the mitochondrial respiratory chain (MRC) as well as the complex I catalytic activity (Coustham et al., 2021) (patent EP23305426.1). The method used is based on *in vitro* data referring to the activity (Heiske et al., 2014) and production (Grivennikova and Vinogradov, 2006; Kussmaul and Hirst, 2006) of ROS in several operating configurations of complex I. These data are introduced in algorithms with normalization to be able to score the future sets of parameters. The molecular behaviour is transcribed with the Michaelis and Menten equations of enzymatic kinetics of complex I reactions. Several models are created with different levels of accuracy, thus increasing the number of parameters to be optimized. After having randomly generated a population of parameters, each solution is passed in a differential evolution algorithm in order to generate an optimized solution population. The models are connected through a cascade structure and are called successively from the simplest model to the most accurate. According to the algorithm, the selected set of parameters can simulate the activity of complex I and the production of ROS at the same time, with results qualitatively and quantitatively close to the input data. Currently, the validation of a set of parameters is based first on the root mean square error,
(1)

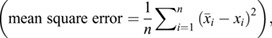
then on the absolute relative error. Finally, human expertise can be used to support these errors in cases in which mathematical artefacts bias the errors and favour a perfectible parameter set. The use of an unequivocal validation tool is one of the ways in which the algorithm can be improved.

We thus addressed the question of the involvement of OPA1 in oxidative metabolism. As haploinsufficiency is primarily responsible for DOA and the effects of OPA1 inactivation are not restricted to RGCs, we assessed the general impact of OPA1 inactivation on oxidative phosphorylation and the redox state by downregulating OPA1 in HeLa cells using an RNA interference strategy. We found cellular respiration to be diminished when OPA1 levels were decreased. This was accompanied by an increase in mitochondrial ROS production, which was buffered by the activation of antioxidant defences, leading to a pro-oxidative state. Our algorithm of complex I is able to simulate its activity, fits with our *in vitro* data and refines the hypothesis for ROS production.

## RESULTS

### OPA1 downregulation affects cellular oxygen consumption and the quantity and activity of MRC complexes

We evaluated the effects of OPA1 downregulation on respiration and glycolysis in HeLa cells, which were transfected with small interfering RNA (siRNA) against OPA1 (siOPA1) using siRNA against Luciferase (siCtrl) as a control. Seventy-two hours after transfection, the quantity of OPA1 was 92% lower in siOPA1-treated cells than in siCtrl cells, but actin levels were not altered (Fig. S1A).

**Fig. 1.**
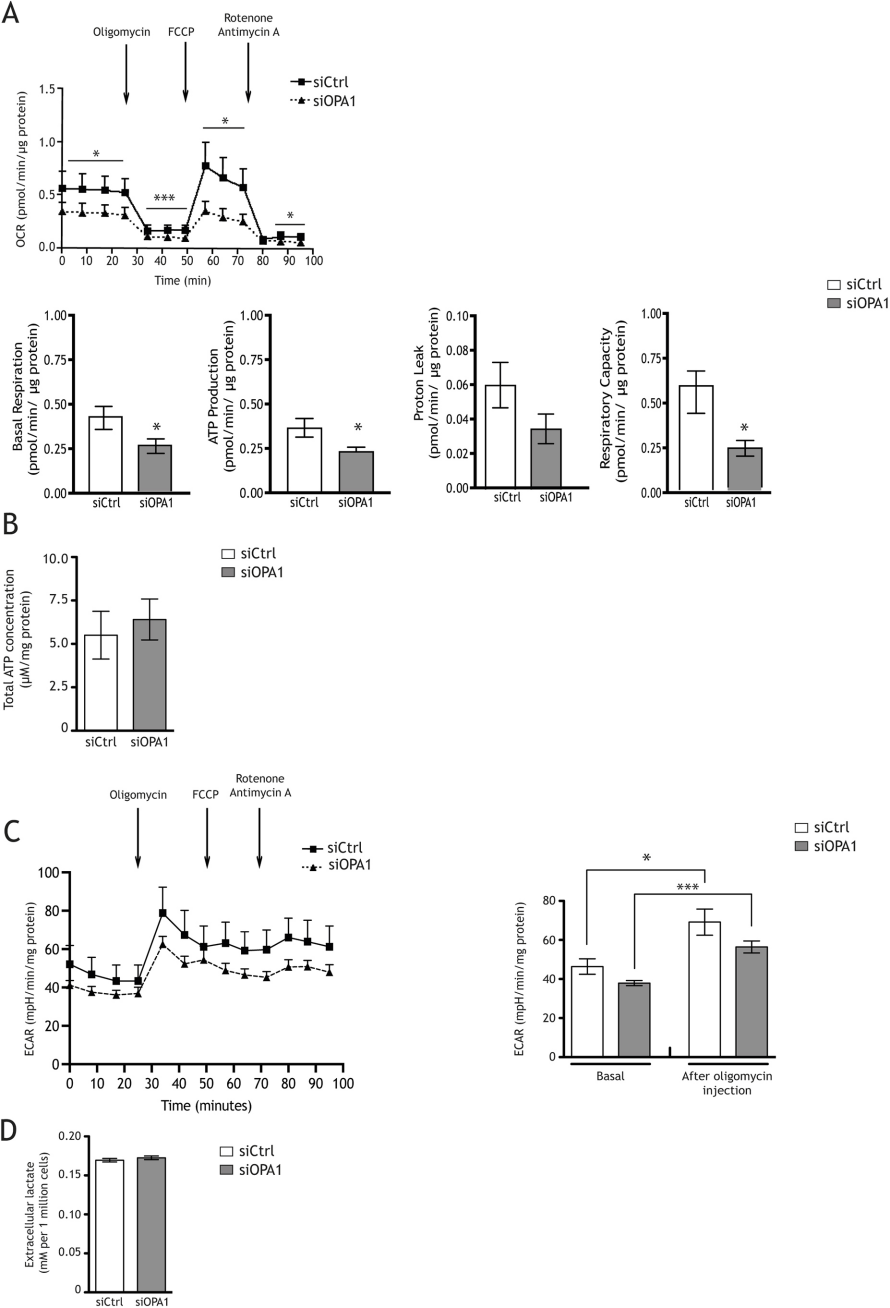
**OPA1 downregulation decreases mitochondrial respiration without changing mitochondrial biomass.** (A) Oxygen consumption rates (OCRs) were measured in siOPA1- and siCtrl-transfected HeLa cells. Spontaneous mitochondrial respiration was significantly lower in siOPA1-transfected cells (0.33±0.02 pmol/min/µg protein) than in control cells (0.53±0.04 pmol/min/µg protein). After oligomycin (1 µM) injection, cell respiration was also significantly lower in siOPA1-transfected cells (0.11±0.01 pmol/min/µg protein) than in control cells (0.17±0.02 pmol/min/µg protein). After FCCP (1 µM) injection, maximal respiratory was significantly lower in siOPA1-transfected cells (0.30±0.04 pmol/min/µg protein) than in control cells (0.67±0.10 pmol/min/µg protein). Finally, rotenone (1 µM) plus antimycin A (1 µM) injection inhibited mitochondrial respiration. Results are expressed as the mean±s.e.m. (*n*=3). Unpaired two-tailed Student's *t*-test (*P<0.05 and ***P<0.001). (B) ATP concentration was unchanged in siOPA1 HeLa cells (grey bar) relative to that in siCtrl-treated (white bar) cells. Results are expressed as the mean±s.e.m. (*n*=3). Paired two-tailed Student's *t*-test. (C) Extracellular acidification rates (ECARs) were unchanged in OPA1-treated cells (basal, 37.93±1.33 pmol/min/µg protein; oligomycin, 56.42±3.08 pmol/min/mg protein) relative to those in siCtrl-treated cells (basal, 46.40±3.93 pmol/min/mg protein; oligomycin, 69.14±6.72 pmol/min/mg protein) under basal conditions and after 1 µM oligomycin injection. As expected, ECAR increased in siCtrl- and siOPA1-transfected cells after oligomycin injection. Results are expressed as the mean±s.e.m. (*n*=3). Unpaired two-tailed Student's *t*-test (*P<0.05 and ***P<0.001). (D) Extracellular lactate was measured by colorimetric analysis in HeLa cells with (grey bar) or without (white bar) siOPA1 (mean±s.e.m., *n*=2). Paired two-tailed Student's *t*-test.

We assessed oxygen consumption rates (OCRs), a direct measure of oxidative phosphorylation activity, and extracellular acidification rates (ECARs), representative of glycolysis, because it accounts for ∼80% of acidification (Wu et al., 2007; Xie et al., 2009), using a Seahorse XF24 flux analyser. Basal respiration was 38% lower in siOPA1-transfected HeLa cells than in siCtrl-treated cells (Fig. 1A). Furthermore, ATP-linked respiration measured in the presence of oligomycin, which inhibits ATP synthase, was reduced by 40%. The maximal OCR measured in the presence of the protonophore carbonyl cyanide 4-(trifluoromethoxy) phenylhydrazone (FCCP), which uncouples oxidation and phosphorylation, was reduced by 58%. The maximal OCR in siOPA1-treated cells was not significantly different from the basal OCR, in contrast to that in siCtrl-transfected cells. Rotenone (complex I inhibitor) and antimycin (complex III inhibitor) considerably decreased the OCR in both siOPA1- and siCtrl-transfected Hela cells, showing that more than 95% of oxygen consumption was due to mitochondrial respiration (Fig. 1A). Of note, the decrease in mitochondrial respiration in siOPA1-treated cells did not correlate with a reduction in mitochondrial biomass. Indeed, citrate synthase activity and quantity, and HSP60, VDAC and TOM20 (also known as TOMM20) levels were unchanged (Fig. S1A,B). We examined the mitochondrial network of siCtrl- and siOPA1-treated Hela cells; the siOPA1-treated cells showed a fragmented mitochondrial network, in contrast to the filamentous mitochondrial network observed in siCtrl-treated cells (Fig. S1C).

Although OPA1 downregulation induced a decrease in mitochondrial respiration, total intracellular ATP levels remained unchanged (Fig. 1B). Furthermore, the decrease in OPA1 levels did not induce a shift towards glycolysis in HeLa cells, as measured by ECAR (Fig. 1C) and extracellular lactate levels in the culture media (Fig. 1D). Similarly, oligomycin treatment increased acidification rates in both siCtrl- and siOPA1-treated HeLa cells (Fig. 1C).

**Fig. 2.**
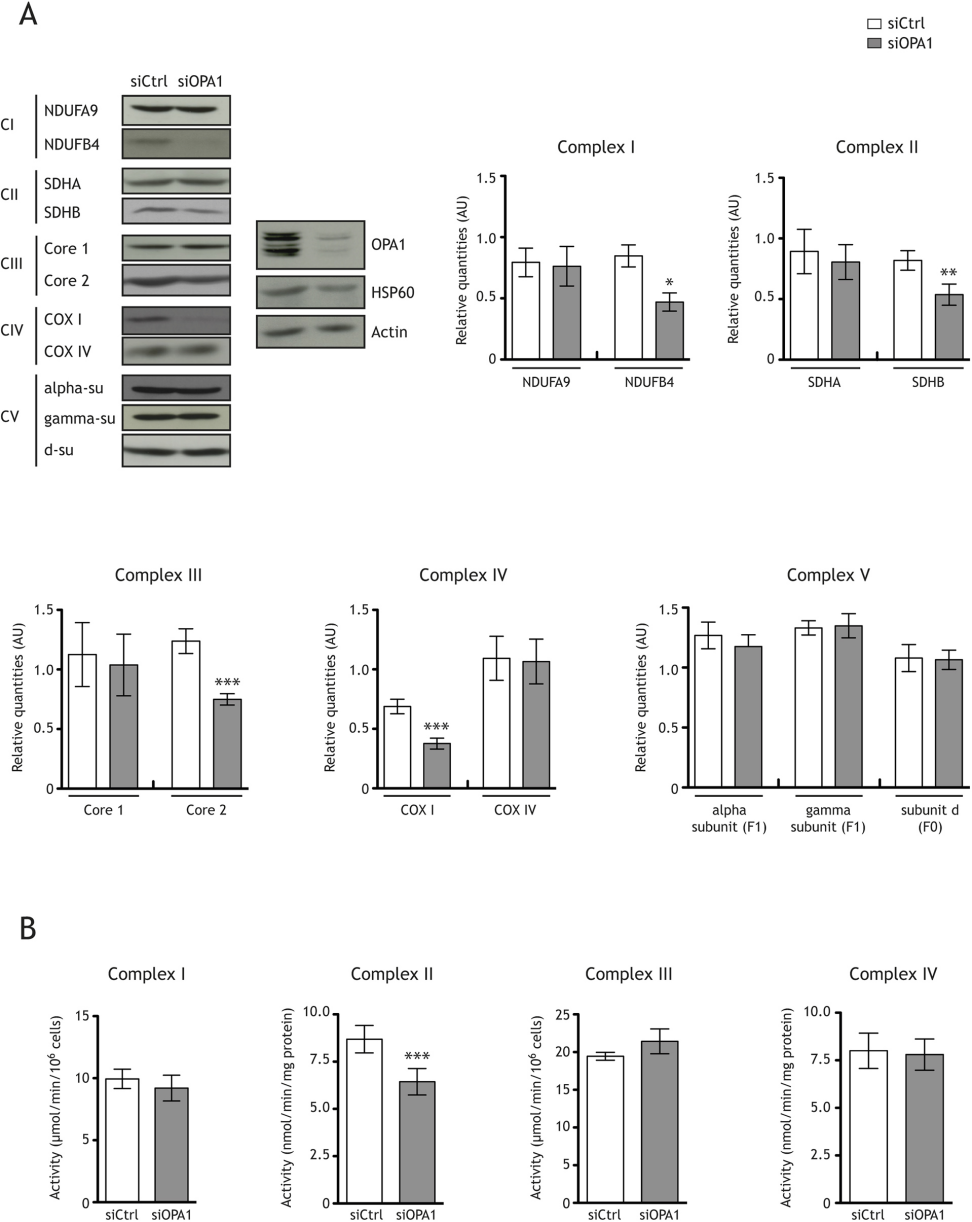
**OPA1 downregulation decreases the level of mitochondrial respiration complex I to IV subunits and complex II activity.** (A) Representative immunoblots and histograms showing the effects of OPA1 downregulation in HeLa cells on the levels of two subunits of the mitochondrial respiratory chain (MRC) complexes [complex I-IV (CI-CIV)] and three subunits (su) of ATP synthase [complex V (CV)] relative to actin. The quantity of NDUFB4 (*n*=10) was significantly lower in siOPA1-transfected HeLa cells [0.47±0.07 arbitrary units (AU)] than in siCtrl-transfected cells (0.85±0.09 AU). The quantity of SDHB (*n*=10) was significantly lower in siOPA1-treated cells (0.68±0.16 AU) than in control cells (0.96±0.16 AU). The level of Core 2 (*n*=10) was significantly lower in siOPA1-treated cells (0.75±0.05 AU) than in control cells (1.238±0.10 AU). The level of COX I (*n*=10) was lower in siOPA1-treated cells (0.37±0.05 AU) than in control cells (0.69±0.06 AU). Paired two-tailed Student's *t*-test (*P<0.05, **P<0.01 and ***P<0.001). (B) Activity of complex I (*n*=3), II (*n*=16), III (*n*=3) and IV (*n*=16) measured *in vitro* in siOPA1- and siCtrl-treated HeLa cells. Succinate dehydrogenase (complex II) activity was lower in siOPA1-treated cells (6.438±0.701 nmol/min/mg protein) than in control cells (8.688±0.7229 nmol/min/mg protein). Results are expressed as the mean±s.e.m. Paired two-tailed Student's (***P<0.001).

The effect of OPA1 downregulation on cellular respiration could be explained by a decrease in the level or activity of MRC complexes. The levels of the subunits NDUFB4 (complex I), SDHB (complex II), Core 2 (complex III) and COX I (also known as MT-CO1; complex IV) were 44%, 29%, 39% and 46% lower, respectively, in siOPA1-treated HeLa cells than in siCtrl-treated cells (Fig. 2A). However, the levels of other subunits, such as NDUFA9 (complex I), SDHA (complex II), Core 1 (complex III) and COX IV (also known as COX4I1; complex IV), were unchanged (Fig. 2A). The levels of three subunits of ATP synthase (alpha and gamma for F1 complex and d for F0 complex) were unaffected by *OPA1* silencing in HeLa cells (Fig. 2A). We then assessed the *in vitro* activities of complexes I to IV by spectrophotometry. Although the activities of complexes I, III and IV were not affected in OPA1-downregulated HeLa cells, complex II activity was reduced by ∼25% (Fig. 2B). Succinate dehydrogenase is also a tricarboxylic acid (TCA) cycle enzyme. We thus assessed the level of NADH, H^+^, the major TCA cycle product, in HeLa cells. The total intracellular level of NADH, H^+^/NAD^+^ was unchanged in siOPA1-treated HeLa cells (Fig. S2). Accordingly, the levels of two other TCA cycle enzyme activities, fumarase and malate dehydrogenase, were also unchanged (Fig. S3C,D).

### OPA1 downregulation results in an imbalanced intracellular redox state

The loss of OPA1 results in impaired mitochondrial respiratory chain function, without disruption of the supply of NADH, H^+^. This can lead to an increase in electron leaks and thus increased ROS production. OPA1 downregulation could therefore lead to an imbalance in intracellular redox homeostasis in HeLa cells.

**Fig. 3.**
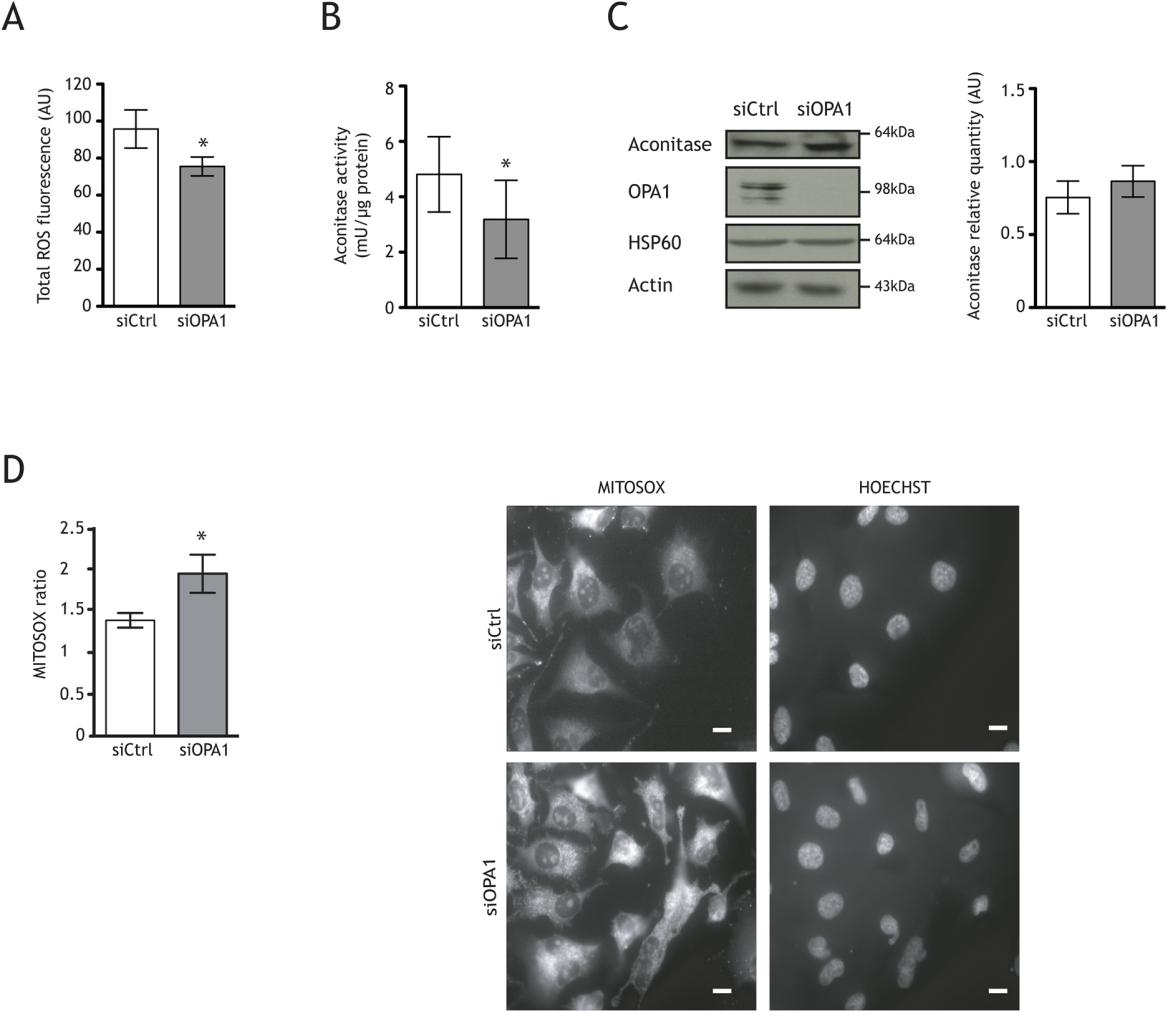
**Imbalanced redox state in OPA1-downregulated cells.** (A) Total reactive oxygen species (ROS) measured by the H_2_-DCFDA probe in siOPA1-transfected HeLa cells (72.63±4.6 AU) was lower than that in siCtrl-treated cells (95.48±9.1 AU). (B) Aconitase activity was lower in siOPA1-treated cells (3.185±1.41 mU/µg protein) than in siCtrl-treated cells (4.811±1.361 mU/µg protein). (C) Representative immunoblots and histograms showing that OPA1 downregulation in HeLa cells has no effect on the expression of aconitase relative to actin. (D) Mitochondrial superoxide production was measured using MitoSOX Red (mitochondria-targeted superoxide indication) with fluorescence microscopy (40×). MitoSOX measurement was performed after 72 h siCtrl (white) or siOPA1 (grey) transfection. Representative histogram of quantitative fluorescence intensity was generated using ImageJ software. The ROS level was higher in siOPA1-tranfected cells (1.9444±0.2278) than in control cells (1.386±0.08754). Representative micrographs of mitochondrial MitoSOX immunocytofluorescence and DNA Hoechst staining in siCtrl- or siOPA1-treated HeLa cells 72 h after transfection. Scale bars: 10 µm. Results are expressed as the mean±s.e.m. [*n*=8 (A), *n*=6 (B), *n*=8 (C) and *n*=3 (D), with more than 200 cells per condition]. Nonparametric test (Mann–Whitney) (A,D) or paired two-tailed Student's *t*-test (B) (*P<0.05).

We investigated this possibility by first measuring total ROS content using an H_2_-DCFDA probe. Surprisingly, ROS levels decreased by 23% in OPA1-downregulated HeLa cells 72 h after transfection (Fig. 3A). We then investigated aconitase activity, which has been shown to be highly sensitive to oxidation due to a damaged FeS core. Inhibition of its activity is thus widely used as a signature of increased mitochondrial ROS production (Gardner et al., 1994; Kelly et al., 2010; Vincent et al., 2005). Aconitase activity was significantly lower (by 34%) in siOPA1-treated HeLa cells than in control cells (Fig. 3B). This decrease could not be attributed to differences in protein quantity, which was unchanged (Fig. 3C). We also used the MitoSOX probe sensitive to mitochondrial ROS and found a significant increase in the signal in siOPA1-transfected cells compared to that in siCtrl-transfected cells (Fig. 3D).

### OPA1 downregulation activates a major antioxidant pathway – the NRF2 transcription factor pathway

Although the total intracellular level of ROS was reduced, the inhibition of aconitase activity provided evidence of an increase in intramitochondrial ROS production. This suggests that intracellular antioxidant responses were activated upon OPA1 downregulation. We thus first analysed the levels of two redox state markers, glutathione and quinone. The ratio between reduced (GSH) and oxidized (GSSG) glutathione in siOPA1-treated HeLa cells increased by 78%, but this increase was not significant (Fig. S3A). The quinone redox state was not altered by the loss of OPA1 (Fig. S3B).

**Fig. 4.**
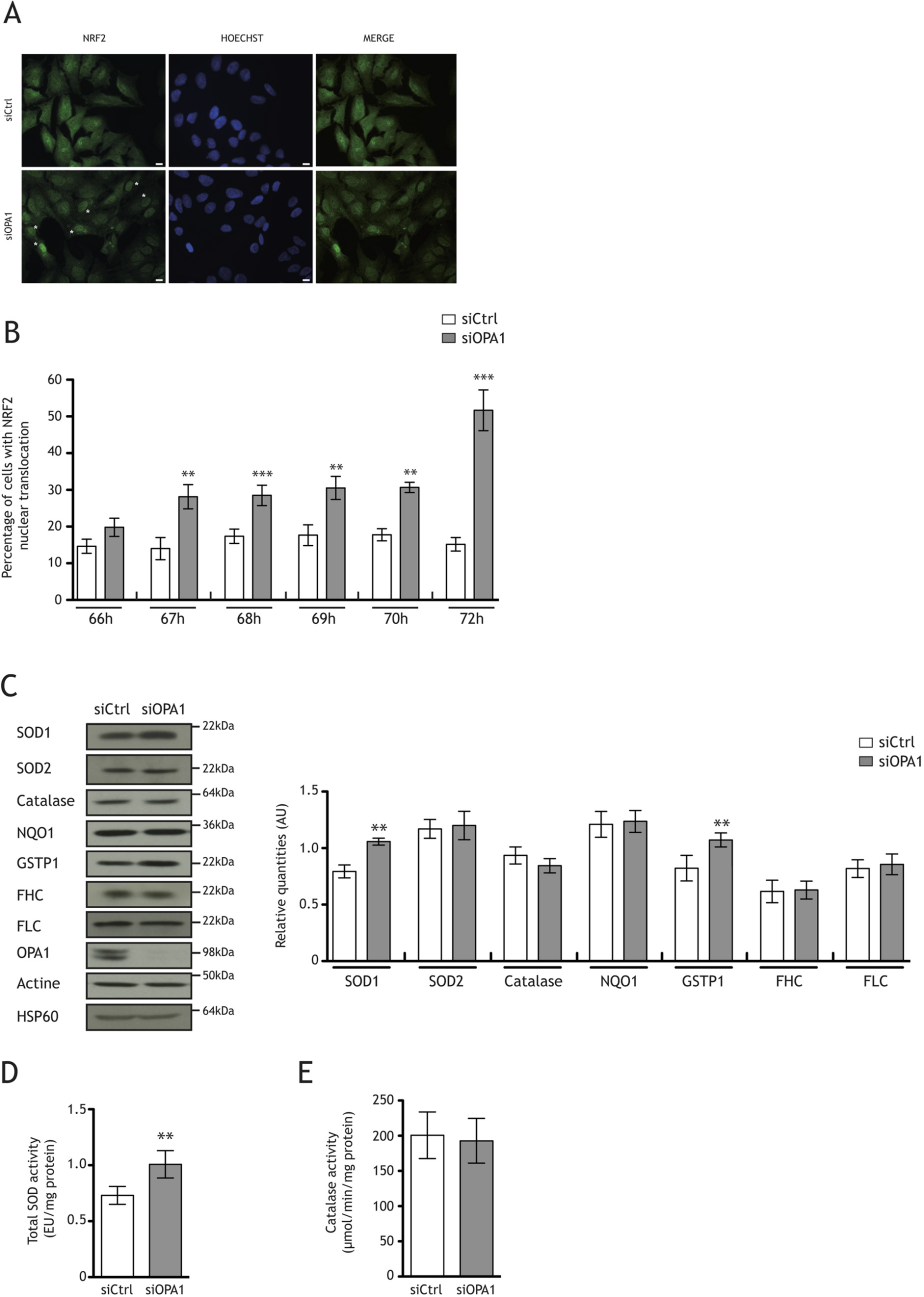
**OPA1 downregulation induces the nuclear translocation of NRF2 and increases the quantity of the NRF2 target proteins SOD1 and GSTP1.** (A) Representative micrographs of NRF2 (green) immunocytofluorescence and DNA Hoechst staining (blue) or merge (green+blue) in siOPA1- or siCtrl-treated HeLa cells 72 h after transfection. Scale bars: 10 μm. (B) Representative histogram showing the percentage of cells with NRF2 nuclear translocation 66, 67, 68, 69, 70 and 72 h after siOPA1 (grey) or siCtrl (white) transfection. Nuclear immunostaining of NRF2 was observed in 15.18±1.86% of control HeLa cells and 51.68±5.57% of siOPA1-treated cells 72 h after transfection. Results are expressed as the mean±s.e.m. [*n*=4-14 (400 cells per condition)]. Nonparametric test (Mann–Whitney test) (**P<0.01 and ***P<0.001). (C) Representative immunoblots and relative quantities of SOD1, SOD2, catalase, NQO1, GSTP1, FHC and FLC protein in siCtrl- and siOPA1-treated HeLa cells. The quantity of SOD1 was higher in siOPA1-treated cells (1.05±0.03 AU) than in control cells (0.79±0.05 AU). The quantity of GSTP1 was higher in siOPA1-treated cells (1.07±0.06 AU) than in control cells (0.82±0.11 AU). Paired two-tailed Student's *t*-test (**P<0.01). (D) Total SOD activity (SOD1 and SOD2) was higher in siOPA1-treated cells (1.01±0.12 EU/mg protein) than in control cells (0.73±0.08 EU/mg protein). Paired two-tailed Student's *t*-test (**P<0.01). (E) Catalase activity was the same in siOPA1- and siCtrl-treated cells. Results are expressed as the mean±s.e.m. [*n*=8 (A), *n*=5 (B) *n*=7 (C) *n*=5 (D) and *n*=7 (E)].

We then investigated the nuclear factor (erythroid-derived 2)-like 2 (NRF2; also known as NFE2L2) pathway, which accounts for a large part of antioxidative responses. We assessed the intracellular localization of NRF2, which indicates its activation, by immunocytofluorescence in HeLa cells from 66 to 72 h post-transfection (Fig. 4A,B). The kinetics of NRF2 immunostaining showed significant NRF2 nuclear relocalization starting 67 h after OPA1 downregulation (Fig. 4B). We already knew that the level of OPA1 protein was reduced by 50% 48 h post-transfection and 90% 72 h post-transfection (data available upon request). We thus hypothesized that NRF2 translocation would occur between 48 and 72 h post-transfection. Seventy-two hours post-transfection, 51% of siOPA1-treated HeLa cells showed NRF2 nuclear localization, whereas only 15% of siCtrl-treated cells showed NRF2 in the nucleus (Fig. 4A,B; Fig. SA4). We thus assessed the levels of several NRF2 target proteins, such as superoxide dismutase 1 (SOD1) and 2 (SOD2), catalase, NQO1, GSTP1, and ferritin heavy (FHC; also known as FTH1) and light (FLC; also known as FTL) chains. The quantity of SOD1 protein was significantly higher (33%) in siOPA1-treated HeLa cells than in control cells (Fig. 4C). This increase correlated with a 37% increase in total SOD (SOD1 and SOD2) activity (Fig. 4D). There was also a 30% increase in the quantity of GSTP1 protein in siOPA1-treated HeLa cells (Fig. 4C). Neither the quantity nor the activity of catalase was altered upon downregulation of OPA1 (Fig. 4C,E). Furthermore, there was no change in NQO1, FHC or FLC protein levels in siOPA1-transfected HeLa cells. The data are summarized in Table 1.

**Table 1.**
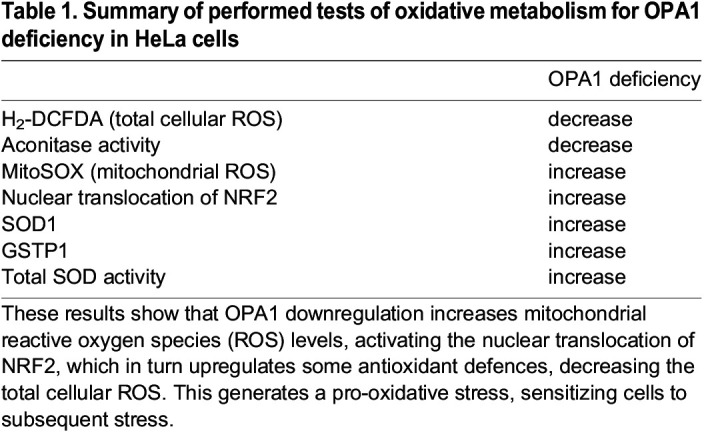
Summary of performed tests of oxidative metabolism for OPA1 deficiency in HeLa cells

### A deterministic mathematical model of complex I is able to predict the results obtained with OPA1 downregulation

**Fig. 5.**
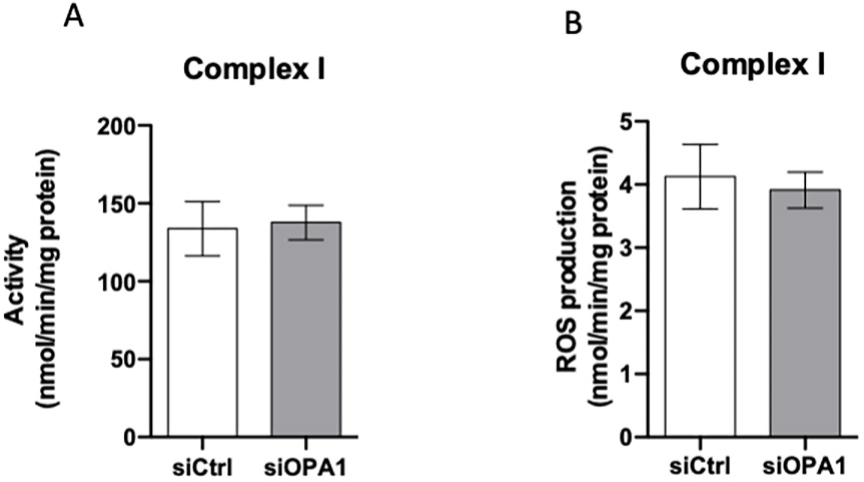
**Our mathematical model simulates complex I activity and ROS production by complex I in the context of siOPA1- or siCtrl-treated cells.** (A) Simulations of complex I activity with the mathematical model in the context of siOPA1 or siCtrl-treated cells. (B) Simulations of ROS production by complex I with the mathematical model in the context of siOPA1- or siCtrl-treated cells.

The data presented in Fig. 5A show that our algorithm fed with data from our analysis did not find any difference in complex I activity while OPA1 was downregulated, or not as well as when we presented *in vitro* activities in Fig. 2B. The algorithm does not express results in the same units as the *in vitro* results of Fig. 2B (μmol/min/mg protein instead of μmol/min/million cells). We verified the stability of the protein content in OPA1-downregulated and wild-type cells (siLuc-treated cells, 99.23±22.56 μg/million cells, *n*=6; siOPA1-treated cells, 77.38±16.20 μg/million cells, *n*=6; *P*=0.4516, unpaired two-tailed Student's *t*-test); as the results are not significantly different, we can compare the two types of results. Moreover, our algorithm presents no differences in ROS production by complex I (Fig. 5B).

## DISCUSSION

Mitochondrial respiration was impaired upon OPA1 downregulation in HeLa cells, as previously shown in several cell types (Millet et al., 2016). This, however, did not trigger a shift towards glycolysis. Overall, the mitochondrial biomass did not change, as estimated by measurement of the levels of several mitochondrial proteins and by citrate synthase activity. However, the level of specific subunits of the first four complexes of the mitochondrial respiratory chain decreased. Therefore, the decrease in mitochondrial respiration measured in siOPA1-treated cells did not correlate with a decrease in the quantity of mitochondria but may be the consequence of impaired activity of the MRC complexes. Only the activity of complex II was decreased.

### Cellular respiration is impaired in OPA1-downregulated cells

OCRs were significantly decreased in OPA1-downregulated cells. This phenomenon, also analysed *in vitro* in OPA1-downregulated neurons, was not accompanied by a shift towards glycolysis (Millet et al., 2016). The decrease in OCR shown in siOPA1-treated cells could not be linked to a decrease in mitochondrial biomass, as the quantities of citrate synthase, HSP60, VDAC and TOM20, and the activity of citrate synthase, remained unchanged in both siCtrl- and siOPA1-treated cells. These results are similar to those in our previous study on OPA1-downregulated rat neurons in primary culture (Millet et al., 2016). In addition, the levels of subunits of the first four complexes of the MRC were found to be similar in OPA1-downregulated HeLa cells and rat neurons *in vitro*, with a marked decrease in the levels of certain subunits. However, the quantity of complex V (ATP synthase) was unchanged upon OPA1 inactivation in both cellular models (Bertholet et al., 2013; Millet et al., 2016). Such a similar mitochondrial metabolism pattern in two very different cell types suggests that OPA1 downregulation triggers mitochondrial dysfunction possibly involved in both neuronal and extra-neuronal degeneration, as seen in DOA+ patients.

### The redox state is imbalanced in OPA1-downregulated cells

A marked decrease in the quantity of MRC subunits and/or dysfunction of the activities of the complexes, while the level of NADH remains stable, results in increased mitochondrial ROS production. The fact that the NADH, H^+^, NAD^+^, and NAD^+^/NADH ratios were unchanged in the siCtrl- and siOPA1-treated HeLa cells implies that the amounts of substrates provided to the MRC were also unchanged in both populations. As already demonstrated in siOPA1-treated rat neurons in primary culture, total intracellular ROS levels decreased. However, aconitase activity also decreased, suggesting an increase in mitochondrial ROS levels. Hence, the increase in intramitochondrial ROS production, which did not correlate with the total intracellular level of ROS, suggests the activation of antioxidant responses upon OPA1 downregulation. We verified this possibility by first analysing two redox state markers, glutathione and quinone. The ratio of reduced (GSH) to oxidized (GSSG) glutathione increased by 121% in siOPA1-treated HeLa cells relative to that in control cells, but was not statistically different (Fig. S3A), and by 78.1% in siOPA1-transfected neurons relative to that in control neurons (*P*<0.05), as previously described (Millet et al., 2016). The quinone redox state [oxidized quinones/(oxidized+reduced quinones)] was not altered by the loss of OPA1 (Fig. S3B). Altogether, these results suggest that OPA1 downregulation activates antioxidant responses by increasing the reduced form of glutathione, the major non-enzymatic antioxidant compound in the cells. Quinones appear not to be involved in mitochondrial ROS detoxification under our conditions. Once again, the similarities between the data obtained with neurons and HeLa cells are striking. These data suggest activation of the antioxidant signalling pathway to buffer the increase in mitochondrial ROS production.

### Antioxidant defences are activated in OPA1-downregulated cells

We analysed the intranuclear level of NRF2, a major transcription factor involved in antioxidant defences (Ma, 2013), in a time-course experiment to assess its activation upon OPA1 downregulation. There was a significant increase in the nuclear translocation of NRF2 in siOPA1-transfected cells from 67 to 72 h post-transfection. Of note, the level of OPA1 decreased by 50% at 48 h post-siOPA1 transfection and by 90% at 72 h post-siOPA1 transfection. This correlation between the decrease in OPA1 content and the nuclear translocation of NRF2 is particularly intriguing, as it underscores the link between OPA1 and the redox state.

We measured the levels of various NRF2 targets in the two conditions; the quantity of SOD1 and GSTP1 increased upon OPA downregulation, as did SOD activity. Similarly, the level and activity of catalase, another NRF2 target, were both elevated in siOPA1-treated neurons (Millet et al., 2016). The detoxification of the superoxide anion was clearly activated in both cellular types (HeLa cells and embryonic cortical neurons in primary culture), underlying the importance of the phenomenon and the universal involvement of OPA1 in redox signalling.

Our study is in accordance with the literature linking OPA1 to the redox state in invertebrates (Kanazawa et al., 2008; Yarosh et al., 2008) and also confirms our results in neurons and transgenic mice cortices (Daloyau et al., 2018; Millet et al., 2016), with better identification of the implicated genes. Moreover, our results underline the ubiquitous effect of OPA1 deficiency on oxidative metabolism in different cell types. A recent study analysing the expression of OPA1 during H_2_O_2_ treatment showed a large decrease in the long form of OPA1 (Garcia et al., 2018). The destabilization of the long form of OPA1 could, in turn, disorganize the inner membrane, emphasizing the effect of the reorganization of the MRC and increasing ROS production. In other words, this phenomenon may represent a vicious circle (Fig. S5).

### Replicative cells as biological material to predict and treat *OPA1* gene mutation-related disorders

The high convergence of the results based on two biological models of DOA that are so strikingly different paves the way towards novel ways of analysing, predicting and possibly treating OPA1 dysfunctions. Moreover, the use of a mathematical model for complex I activity and ROS production is interesting. Our algorithm simulates perfectly the complex I activity whether or not there is OPA1 expression. The activities in *in silico* simulations (Fig. 5A) and *in vivo* analysis (Fig. 2B) are stable in both cases. In terms of ROS production, the simulations suggest that complex I is not the principal ROS producer. In this context, complex III would be the most probable effector of the increase in mitochondrial ROS production. *In silico* simulations with our mathematical model of complex I activity combined with ROS production will become a powerful tool to integrate different biological data and to predict the evolution of the described processes.

This is a real proof of concept for translational medicine in the area of neurodegenerative diseases. We plan to analyse the oxidative metabolism of DOA and DOA+ patients using fibroblasts from patient biopsies (Millet et al., 2016) and epithelial cells to predict the evolution of the disease, which is currently impossible to control. We will combine experimental and mathematical models to enhance the power of wet-lab investigations and more accurately predict the evolution of DOA and *OPA1* gene-related disorders by considering mitochondrial ROS production and the detoxifying pathways of ROS (Merabet et al., 2017; Millet et al., 2017).

Mitochondria have long been proposed to play a key role in ageing (Green et al., 2011). As a consequence of their central role in ATP formation via the MRC, mitochondria are the major source of ROS and are thus highly involved in oxidative stress processes (Brookes et al., 2004). However, mitochondria are also targets of these molecules (Brookes et al., 2004). Under physiological conditions, ∼1-3% of molecular oxygen is incompletely reduced during redox reactions in the MRC, leading to the production of ROS superoxide anion (O2^.−^) by-products. In this scenario, complex interactions in antioxidant defence systems repress oxidative stress within mitochondria (Kienhofer et al., 2009). Cellular systems that protect against oxidants involve antioxidant defence enzymes [SOD, glutathione peroxidase (GPX) and catalase] (Kienhofer et al., 2009), oxidant scavengers (vitamin E, vitamin C, carotenoids, uric acid and polyphenols), and mechanisms that repair oxidant-induced damage to lipids, proteins or DNA. Despite these protective mechanisms, the uncontrolled generation of ROS can overwhelm the capacity of antioxidant protection, causing mitochondrial dysfunction (for example, Parkinson disease directly involves complex I dysfunction). *In vivo* studies in transgenic mice have shown that the overexpression of catalase targeted to mitochondria reduces age-associated diseases and increases lifespan (López-Armada et al., 2013; Schriner et al., 2005). Based on these observations, our entire body of work suggests that the downregulation of OPA1 in cellular and animal models induces an imbalance in the redox state, which could lead to premature cellular ageing.

## MATERIALS AND METHODS

### Cell culture

HeLa cells from the American Type Culture Collection (Manassas, VA, USA) were cultured in Dulbecco's modified Eagle's medium with 4.5 g/l glucose (DMEM; Invitrogen), supplemented with 10% foetal calf serum, penicillin (100 units/ml) and streptomycin (100 mg/ml), in an incubator at 37°C and 5% CO_2_. HeLa cells were electroporated using a Cell Line Kit R (Amaxa, Lonza) with 1.5 µg control siRNA (D-001210-02, Dharmacon Research) or human OPA1 siRNA (D-005273-03, target sequence AAAGAAGGCUGUACCGUUA, Dharmacon Research) per 10^6^ cells.

### Measurement of OCRs, ECARs and ATP levels

OCRs and ECARs were measured using an XF24 Extracellular Flux Analyser (Seahorse Bioscience, North Billerica, MA, USA). HeLa cells (15×10^3^) transfected with siCtrl or siOPA1 were plated on XF24 microplates 3 days before OCR measurements. Dual-analyte sensor cartridges were soaked in XF Calibrant Solution (Seahorse Biosciences) in 24-well cell-culture microplates overnight at 37°C to hydrate. Approximately 1 h prior to experimentation, injection ports on the sensor cartridge were filled with oligomycin (1 µM), FCCP (1 µM), and rotenone (1 µM) plus antimycin A (1 µM). Plates were then loaded into the XF24 instrument for calibration. For measuring oxygen consumption, the DMEM of HeLa cells was replaced by DMEM supplemented with NaCl (143 mM), Phenol Red (3 mg/ml), glucose (10 mM), glutamine (2 mM) and pyruvate (2 mM) at pH 7.4, and the plates were maintained at 37°C 1 h prior to experimentation. Plates were then loaded into the Seahorse XF24 analyser following the manufacturer's instructions.

ATP measurements in HeLa cells were determined using an ATP Colorimetric/Fluorometric Assay kit (Abcam). Intracellular ATP levels were determined using the colorimetric assay, following the manufacturer's instructions, on 1.10^6^ HeLa cells transfected with control siRNA or OPA1 siRNA. ATP content was measured in duplicate (570 nm) and calculated per microgram of protein.

### *In vitro* activities of respiratory chain complexes

The activities of respiratory complexes II and IV and citrate synthase were measured as previously described (Agier et al., 2012). The activities of respiratory complexes I and III were measured as previously described (Spinazzi et al., 2011).

### Determination of fumarase activity

The reaction medium consisted of 100 mM KPO_4_ pH 7.4, 5 mM malate, 60 μg cell protein. In a measuring vessel, 60 μg cell protein, previously homogenized, was added to 930 μl reaction medium. After calibration of the reading at 250 nm, at 37°C, a baseline was read for 5 min, then the reaction was triggered by adding 50 μl of 1 M malate in 500 mM KPO_4_ pH 7.4. Specific activity was calculated in nmol/min/mg protein using the extinction coefficient for fumaric acid (ε=2.44).

### Determination of malate dehydrogenase activity

The reaction medium consisted of 50 mM sodium phosphate, 5 mM trisodium DL-isocitrate and 60 μg cell protein. In a measuring vessel, 60 μg cellular protein, previously homogenized, was added to 965 μl reaction medium. After calibration of the reading at 240 nm, at 37°C in air, a baseline was read for 5 min, then the reaction was triggered by adding 5 μl of 1 M isocitrate. Specific activity was calculated in nmol/min/mg protein using the extinction coefficient for isocitrate (ε=3.6).

### Extracellular lactate levels

Extracellular lactate levels were measured using a colorimetric assay (BioMérieux). Lactate was measured in media supernatants (1:10 dilution) at 505 nm 72 h after transfection of HeLa cells with siCtrl or siOPA1, following the manufacturer's instructions.

### Immunoblot analysis

Transfected HeLa cells were lysed for 30 min in a RIPA buffer containing 50 mM Tris-HCl pH 7.5, 250 mM NaCl, 5 mM EDTA, 5 mM EGTA, 1 mM dithiothreitol, 0.1% Triton X-100, 0.1% SDS, 1% deoxycholate, 1% NP40 and protease inhibitors (cOmplete Protease Inhibitor Mixture, Roche Applied Science). Cell lysates were then centrifuged at 14,000 ***g*** at 4°C for 10 min. The total protein concentration was determined in the supernatant using the Bradford protein assay (Bio-Rad).

Proteins (100-200 μg) were separated by SDS-PAGE (8-15%) and transferred onto nitrocellulose membranes (Whatman, Protran). Free binding sites were blocked with 5% non-fat dry milk in 1× Tris-buffered saline pH 7.6 containing 0.2% Tween 20 (blocking buffer). The membranes were probed with various primary antibodies [anti-OPA1 (1:300; 612607, BD Biosciences), anti-actin (1:25,000; MAB1501, Chemicon), anti-HSP60 (1:8000; H-3524, Sigma-Aldrich), anti-OXPHOS (1:200; MS604, Mitosciences), anti-NDUFB4 (1:500; MS107, Mitosciences), anti-NDUFA9 (1:100; 459100, Mitosciences), anti-SDHA (1:1000; ab14715, Abcam), anti-Core 1 (1:500; Invitrogen), anti-COX IV (1:250; 4750, Cell Signaling Technology), anti-ATP5C1 (1:500; AP9239a, Abgent), anti-ATP5H (1:5000; ab110275, Abcam), anti-aconitase (1:500; ab83528, Abcam), anti-SOD1 (1:2000; 2018-5, Epitomics), anti-SOD2 (1:2000; 2299-5, Epitomics), anti-catalase (1:3000; ab16731 Abcam), anti-NQO1 (1:3000; ab28947, Abcam), anti-GSTP1 (1:8000; GS72, Oxford Biochemical Research), anti-ferritin heavy chain (1:500; ab81444, Abcam) and anti-ferritin light chain (1:4000; ab69090, Abcam)] and incubated overnight at 4°C in blocking buffer. After chemiluminescent detection of horseradish peroxidase-conjugated secondary antibody (1:50,000; ab6789, Abcam), scanned photographic films were analysed using ImageJ software.

### Immunocytochemistry or MitoTracker staining

HeLa cells were fixed for 20 min with PBS containing 3.7% formaldehyde, permeabilized for 5 min in 1× PBS, 0.25% Triton X-100 and incubated for 10 min at −20°C with methanol prior to nuclear NRF2 detection. Non-specific binding sites were blocked with 3% bovine serum albumin in 1× PBS for 15-30 min at room temperature. Cells were immunostained with rabbit polyclonal anti-NRF2 antibody (1:50; sc-13032 Santa Cruz Biotechnology) for 1 h at 37°C. HeLa cells were then incubated with Alexa Fluor 488-conjugated secondary antibodies (1:300; Molecular Probes), labelled with 0.25 µg/ml Hoechst in 1× PBS for 5 min and mounted in Mowiol. Immunolabelling of HeLa cells 66, 67, 68, 69, 70 and 72 h after siCtrl or siOPA1 transfection was visualized under a fluorescence microscope (Nikon Eclipse 80i), and the images were acquired using an NIS-Element (Nikon Digital Sight DUS2 camera). HeLa cells showing accumulation of NRF2 staining in the nucleus were counted by stack with Hoechst labelling of the nucleus using ImageJ software.

The mitochondrial network was stained with MitoTracker Red (M22425, Invitrogen) in culture medium for 15 min at 37°C and 5% CO_2._ Then, cells were fixed for 20 min with PBS containing 3.7% formaldehyde, permeabilized for 5 min in 1× PBS, 0.25% Triton™ X-100 and mounted in DAPI-mounting medium (VECTASHIELD With DAPI, H-1200-10, Eurobio Scientific).

### Measurement of ROS levels and aconitase activity

ROS levels in HeLa cells were measured using the fluorescent dye 2′,7′-dichlorodihydrofluorescein diacetate (H_2_-DCFDA, Molecular Probes) at 4 µM for 30 min at 37°C. Fluorescence intensities were measured at 493 nm in a WALLAC VICTOR 1480 Multilabel Counter.

Mitochondrial superoxide production was measured using MitoSOX Red (Thermo Fisher Scientific) at 5 µM according to the manufacturer's protocol with fluorescence microscopy (40×). MitoSOX measurement was performed after 72 h siCtrl or siOPA1 transfection. Fluorescence intensity was quantified using ImageJ software.

Aconitase activity in HeLa cells was measured at 525 nm (UVIKON Spectrophotometer 922, Perkin Elmer) using a protocol described in Colombani et al. (2009).

### Enzymatic antioxidant activities

SOD activities (MnSOD and Cu/ZnSOD) were assayed by inhibition of pyrogallol auto-oxidation in HeLa cell extracts, as performed for aconitase activity. One enzymatic unit of SOD activity was defined as the amount of enzyme that inhibited pyrogallol auto-oxidation by 50% (Galinier et al., 2006). Catalase activity was determined by measuring the decomposition of H_2_O_2_ at 240 nm (Galinier et al., 2006).

### Statistical analysis

Most statistical analysis was performed using paired two-tailed Student's *t*-test because of the systematic comparison between siCtrl- and siOPA1-treated HeLa cells. Differences between OCRs in siCtrl- and siOPA1-treated HeLa cells were investigated using a nonparametric test (Mann–Whitney). *P*<0.05 was considered significant.

### Patents

Two patents resulting from the work reported in this paper have been published: PCT/EP2015/056814 (27 March 2015) and EP14305448 (27 March 2014).

## Supplementary Material

10.1242/dmm.050266_sup1 Supplementary information
