# Correction: OPA1 deficiency impairs oxidative metabolism in cycling cells, underlining a translational approach for degenerative diseases

**DOI:** 10.1242/dmm.050815

**Published:** 2024-04-26

**Authors:** Aurélie M. C. Millet, Corentin Coustham, Camille Champigny, Nadege Merabet, Marlène Botella, Christine Demeilliers, Anne Devin, Anne Galinier, Pascale Belenguer, Joel Bordeneuve-Guibé, Noélie Davezac

There were errors in *Dis. Model. Mech.* (2023) **16**, dmm050266 (doi:10.1242/dmm.050266).

The units for aconitase activity in Fig. 3B should be mU/mg protein rather than mU/μg protein.

One of the patents (EP23305426.1) cited in the Introduction is still pending, which was not stated in the original paper. The text in the third paragraph of the Introduction describing the mathematical model on ROS production of complex I of the mitochondrial respiratory chain (MRC) and complex I catalytic activity has been updated to reflect this.

Also, all authors agreed to add Nadege Merabet to the author list for her input on the method used to develop part of the model used in Fig. 5.

These issues have been corrected in the online and PDF versions of the paper. The authors apologise for these errors and any inconvenience caused.

