# Supplementary material for: OPA1 deficiency impairs oxidative metabolism in cycling cells, underlining a translational approach for degenerative diseases
Source: Dis Model Mech. 2023 Sep 20;16(9):dmm050266. doi: 10.1242/dmm.050266 (PMC10538295; doi:10.1242/dmm.050266)
Supplement: Supplementary information [file dmm-16-050266-s1.pdf]

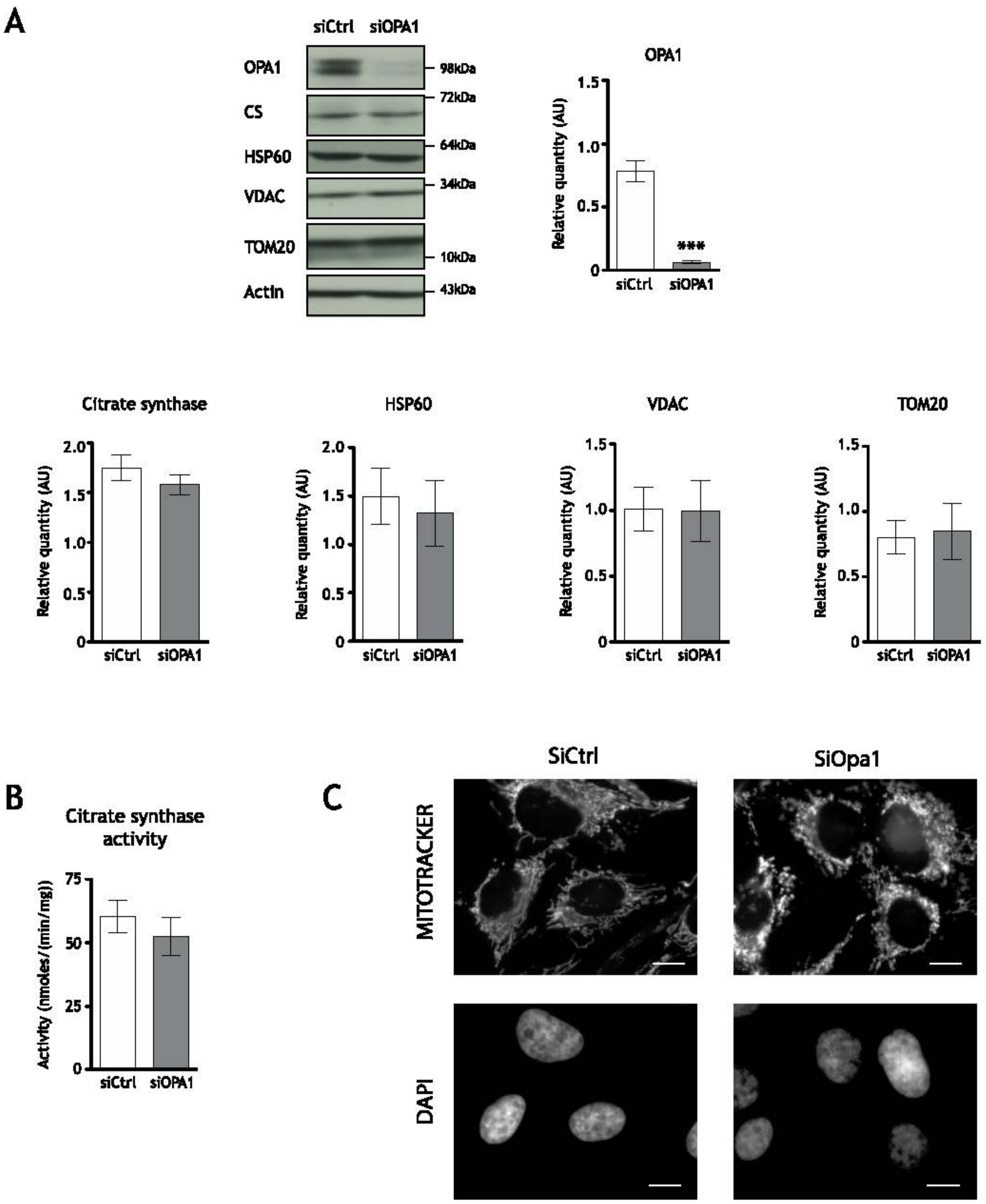
A
siCtrl
siOPA1
OPA1
CS
HSP60
VDAC
TOM20
Actin
98kDa
72kDa
64kDa
34kDa
10kDa
43kDa
OPA1
Relative quantity (AU)
***
siCtrl
siOPA1
Citrate synthase
HSP60
VDAC
TOM20
B
Citrate synthase activity
Activity (nmoles/(min/mg))
C
SiCtrl
SiOpa1
MITOTRACKER
DAPI

Fig. S1. Mitochondrial biomass is not affected by the loss of OPA1. (A) Representative immunoblots and histograms showing protein levels of OPA1 (inner membrane), citrate synthase (matrix), HSP60 (matrix), VDAC (outer membrane) and TOM20 (outer membrane) relative to actin, in OPA1 down-regulated cells and control cells. OPA1 protein level is drastically decreased in siOPA1-transfected HeLa cells (92%). In OPA1 down-regulated HeLa cells, there are no differences of all protein quantities compared to control cells. **(B)** Citrate synthase activity, a TCA cycle enzyme, is unchanged in both conditions. Results are expressed as mean +/- SEM with n=8 (A), n=16 (B). P values were determined by Student's-paired t-test p<0.001***. (C) The mitochondrial network in siOPA1-transfected HeLa cells is punctuated and filamentous in siCtr-transfected HeLa cells. Nuclei are stained with DAPI and the mitochondrial network is coloured with Mitotracker. Scale bar represents 10 µm.

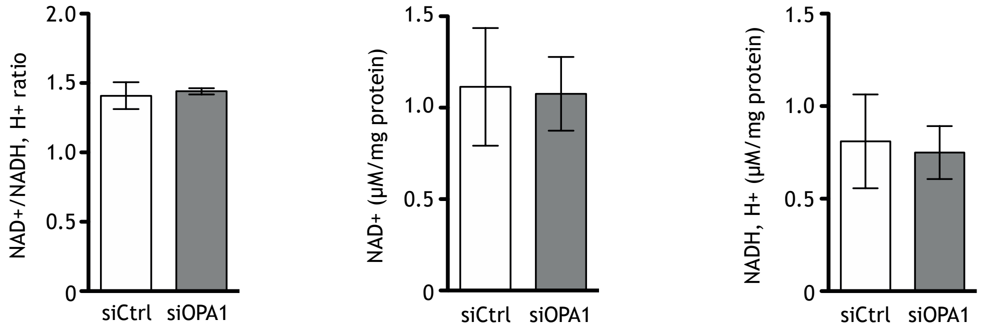

Fig. S2. Down regulation of OPA1 doesn't change NADH and NAD^+ intracellular levels. Ratio of NAD^+/NADH, H^+ and total intracellular levels of NAD^+ and NADH, H^+ were unchanged in HeLa cells transfected with siOPA1, when compared to siCtrl cells. Results are expressed as mean +/- SEM n=5. P values were determined by Student's-paired t-test.

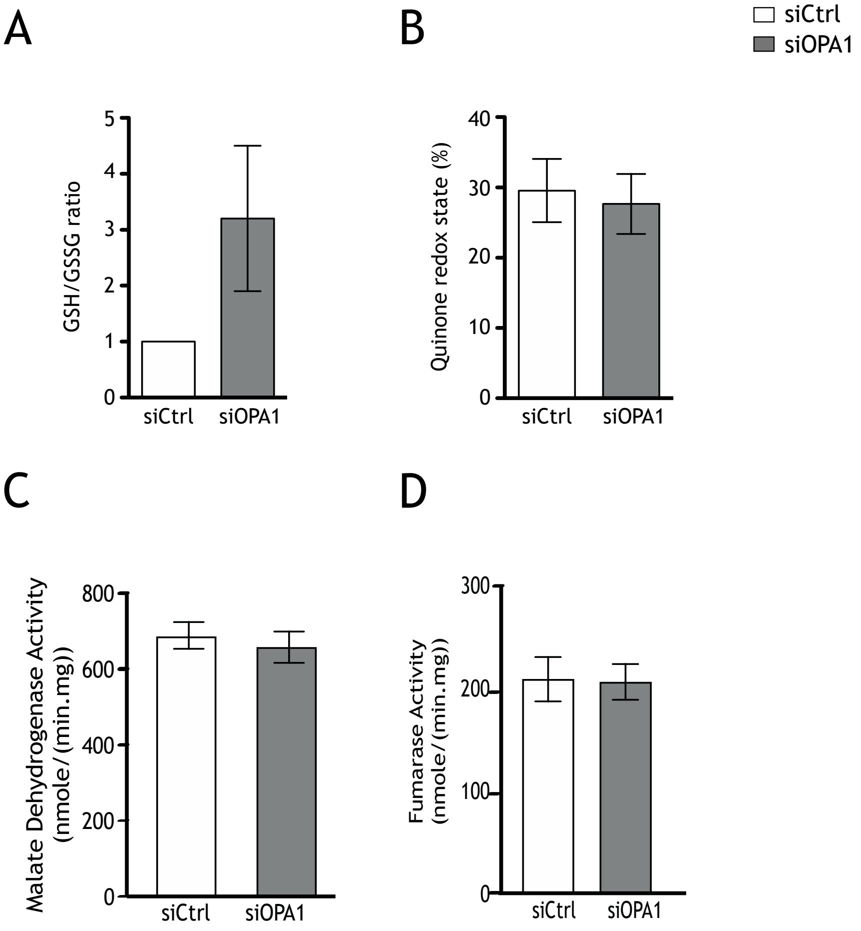

Fig. S3. Glutathione reduced form is increased in OPA1 down-regulated cells. (A) Reduced (GSH) and oxidized (GSSG) glutathione levels are measured in HeLa cells transfected with siOPA1 (grey), compared to control cells (white). **(B)** Oxidized and reduced quinones are evaluated. Quinone redox state is the ratio of oxidized form of quinone on total forms. **(C)** Malate Dehydrogenase activity is evaluated. **(D)** Fumarase activity is evaluated. Results are expressed as mean +/- SEM n=4 (A), n=8 (B), n=18 (C), n=16 (D). P values were determined by Student's-paired t-test.

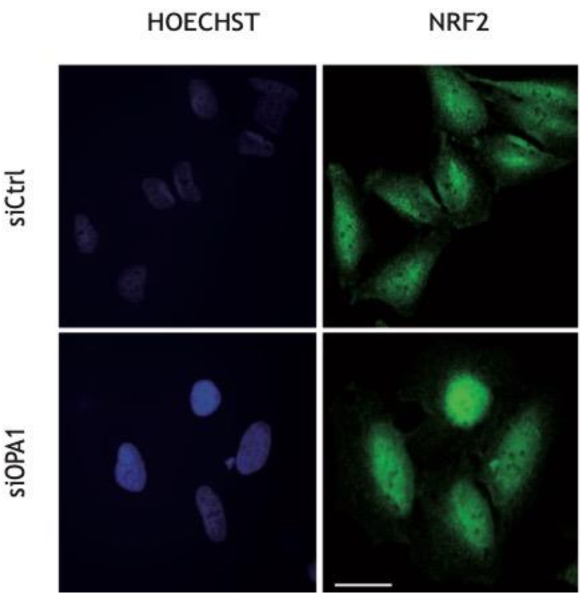

Fig. S4. Magnification of NRF2 nuclear translocation in SiOPA1 treated cells. Representative micrographs of NRF2 (green) immunocytofluorescence and DNA Hoechst staining (blue) in siOPA1- or siCtrl-treated HeLa cells 72 h after transfection. (The scale bar represents 5 μm)

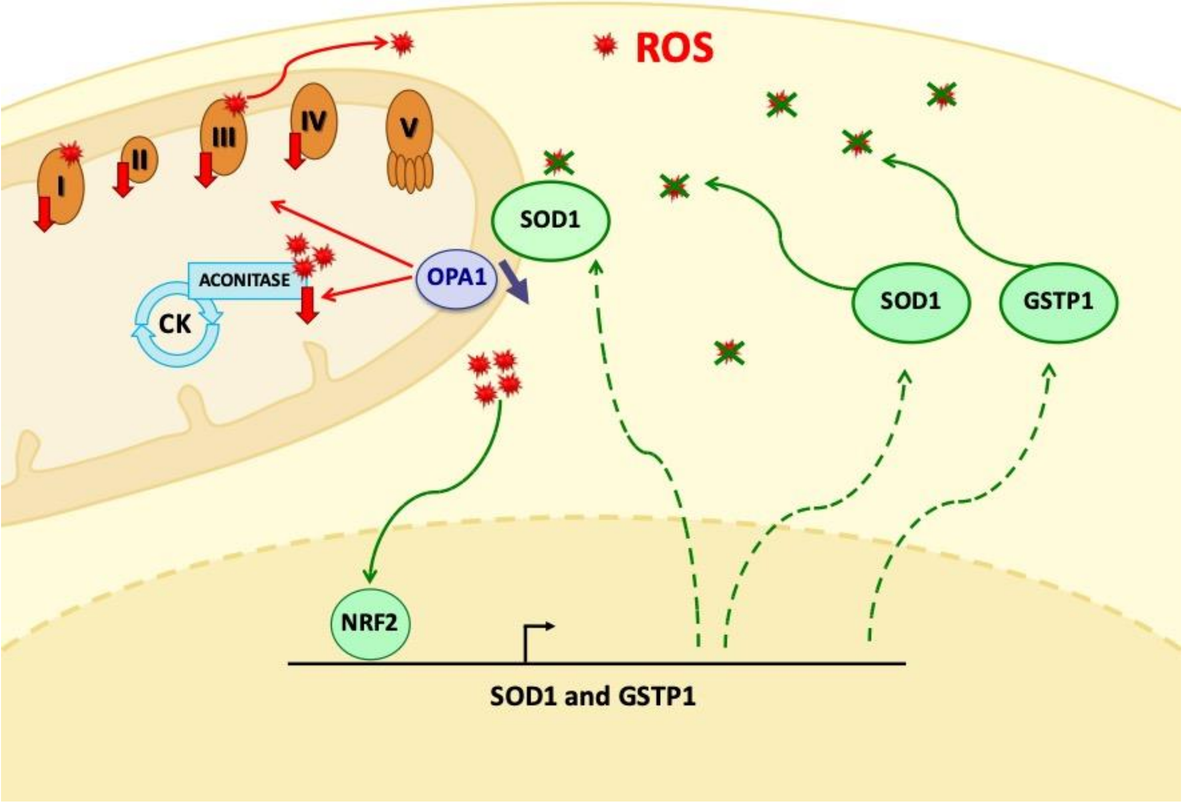

Fig. S5. Graphical Abstract. In OPA1 depleted cells, some subunits of the the first four complexes of the Mitochondrial Respiratory Chain are decreased without altered total ATP production. The cells fall in a pro-oxidative state as underlined by the decreased aconitase activity, the NRF2 transcription factor which is translocated into the nucleus and the GSTP1 and SOD1 protein quantities that are significantly increased and in turn decrease the intra-cellular ROS levels.

Disease Models & Mechanisms • Supplementary information
